# Molecular Techniques for MTBC and NTM Differentiation: Diagnostic Accuracy of STANDARD™ M10 MTB/NTM and Potential Applications

**DOI:** 10.3390/diagnostics16040594

**Published:** 2026-02-16

**Authors:** Mattia Genco, Silvia Alizzi, Paolo Valesella, Anna Camaggi, Marco Iannaccone, Valeria Allizond, Giuliana Banche, Alessandro Bondi, Maria Simona Caroppo, Rocco Francesco Rinaldo, Paolo Solidoro, Silvia Corcione, Andrea Calcagno, Antonella Rossati, Cristina Costa, Antonio Curtoni

**Affiliations:** 1 Department of Public Health and Paediatrics, University of Turin, 10126 Turin, Italy; mattia.genco@unito.it (M.G.); giuliana.banche@unito.it (G.B.); antonio.curtoni@unito.it (A.C.); 2 Microbiology and Virology Unit, University Hospital Città della Salute e della Scienza di Torino, 10126 Turin, Italy; salizzi@cittadellasalute.to.it (S.A.); pvalesella@cittadellasalute.to.it (P.V.); miannaccone@cittadellasalute.to.it (M.I.); 3 Microbiology and Virology Unit, Azienda Ospedaliero-Universitaria Maggiore della Carità di Novara, 28100 Novara, Italy; anna.camaggi@maggioreosp.novara.it (A.C.); msimona.caroppo@maggioreosp.novara.it (M.S.C.); 4 Division of Respiratory Medicine, Cardiovascular and Thoracic Department, University Hospital Città della Salute e della Scienza di Torino, 10126 Turin, Italypaolo.solidoro@unito.it (P.S.); 5 Medical Sciences Department, University of Turin, 10126 Turin, Italy; 6 Tufts Medical Center, School of Medicine, Tufts University, Boston, MA 02111, USA; 7 Department of Translational Medicine, University of Eastern Piedmont, 28100 Novara, Italy; 8 SCDO Malattie Infettive, Ospedale “Maggiore della Carità”, 28100 Novara, Italy; 9 Stop TB Italy, 20159 Milan, Italy

**Keywords:** *Mycobacterium tuberculosis* complex, non-tuberculous mycobacteria, fluorescence microscopy, culture, GeneXpert MTB/RIF Ultra, STANDARD™ M10 MTB/NTM

## Abstract

**Background.** Over the past decade, the World Health Organization has highlighted the need for rapid molecular diagnostics as first-line tools for detecting *Mycobacterium tuberculosis* complex (MTBC) to strengthen global tuberculosis control. At the same time, infections caused by non-tuberculous mycobacteria (NTM) have become increasingly prevalent, particularly in low TB-burden countries such as Italy. This changing epidemiological scenario underscores the necessity for fast and reliable methods capable of distinguishing NTM from MTBC, a critical step for guiding appropriate treatment. This study evaluated the diagnostic accuracy and potential applications of the STANDARD™ M10 MTB/NTM assay, which simultaneously detects and differentiates MTBC and NTM. **Methods.** A total of 155 clinical specimens (78.1% respiratory) from patients with suspected mycobacterial infection were tested by fluorescence microscopy, GeneXpert MTB/RIF Ultra (respiratory samples only), STANDARD™ M10 MTB/NTM and culture, used as the reference method. **Results.** Culture detected MTBC in 54% and NTM (predominantly slow-growing species) in 46% of samples. STANDARD™ M10 showed overall sensitivity and specificity of 70% and 100%, respectively. For MTBC, sensitivity was 85.1% with almost perfect agreement with culture (κ = 0.866), while for NTM, sensitivity was 50% with moderate agreement (κ = 0.566). Sensitivity decreased in microscopy-negative/culture-positive specimens, particularly for NTM. Compared with GeneXpert MTB/RIF Ultra, STANDARD™ M10 exhibited slightly lower sensitivity for MTBC but retained excellent specificity. **Conclusions**. STANDARD™ M10 MTB/NTM represents a rapid, fully automated tool to support early etiological diagnosis and MTB/NTM differentiation, mainly in selected samples or high-risk patients, but it does not replace culture or molecular tests providing species identification and MTBC drug-resistance profiling.

## 1. Introduction

Among the genus *Mycobacterium*, with the exception of *M. leprae* and *M. lepromatosis*, the clinically relevant species belong to the *M. tuberculosis* complex (MTBC), or to the group of non-tuberculous mycobacteria (NTM). The MTBC comprises small acid-fast bacilli and includes nine different species, and *M. tuberculosis* is the principal etiological agent of tuberculosis (TB). TB remains one of the more relevant causes of infectious disease morbidity and mortality globally, notwithstanding significant advances in diagnosis and treatment over the last few decades [1,2]. It is typically an airborne disease transmitted by infected individuals through respiratory secretions. Following exposure to the pathogen, in case of infection (20–25%), TB can progress with clinical manifestations (active TB, 1–2.5%) or remain asymptomatic in the latent (LTBI) form for decades, after which it may reactivate, generally causing signs and symptoms. Notably, both active and subclinical TB are responsible for *MTBC* spreading to other subjects [3,4,5,6]. Generally, TB affects the lungs, specifically parenchyma or the tracheobronchial tree, but it can also spread to other organs—such as kidneys, spine, and brain—causing extrapulmonary TB [2,7].

The World Health Organization (WHO), during 2023, estimates that TB affected about 10.8 million individuals worldwide and resulted in 1.25 million deaths, even if these results might be underestimated due to undiagnosed cases that accounted for approximately 24% [7,8]. Despite the epidemiology of TB differing by region, there are burden countries—sub-Saharan Africa and parts of Asia—that are responsible for a significant proportion of global cases [9,10]. In contrast, the TB incidence in Italy has been gradually decreasing, with 5–6 cases per 100,000 population, which is below the European average, and regions in Northern Italy are more heavily affected than those in the South, highlighting the need for continued surveillance and control efforts [11]. Moreover, the escalation of multidrug-resistant tuberculosis (MDR-TB) and extensively drug-resistant tuberculosis (XDR-TB) has further complicated the fight against the disease [5,12]. Long-term observation, accurate laboratory analysis, and correct treatment strategies are critical for controlling TB transmission and reducing the associated burden [1,5,13].

Another challenging issue in Public Health is represented by NTM. This group includes over 200 species of mycobacteria, opportunistic environmental pathogens contaminating water and soil that can cause both pulmonary and extrapulmonary diseases [14,15,16]. The NTM-disease burden is increasing globally due to multiple factors, including enhanced awareness, improved diagnostic tools, and, most of all, the rise in its major risk factors [17,18,19]. In low TB-burden countries, a relevant proportion of patients investigated for suspected tuberculosis are eventually diagnosed with non-tuberculous mycobacterial disease, highlighting the clinical importance of rapid and accurate MTBC/NTM differentiation [17]. Population aging, along with related comorbidities, respiratory disorders, and immunosuppressive therapies, is, in fact, becoming a common feature, especially in populations of high-income countries [17,18].

NTM disease incidence significantly varies according to epidemiological heterogeneity, clinical presentation, diagnostic criteria, and notification policies. The majority of available data pertains to the NTM-pulmonary disease (NTM-PD). In the United States, the annual average incidence was 20.1 per 100,000 population. In Europe, NTM-PD is not a widespread notifiable disease, and epidemiological data are available for only a few countries (e.g., in the UK, the incidence ranges from 6.5 to 7.6 cases per 100,000 inhabitants) [17,18,19].

As regards mycobacterial infections, laboratory diagnosis relies on a combination of immunological and microbiological methods. Indirect immunological tests, such as the tuberculin skin test and interferon-γ release assays (IGRAs), are used to detect previous infection with the MTBC, but they are not applicable to NTM disease [20,21]. These tests identify a host immune response to MTBC exposure; however, their main limitation is the inability to differentiate between latent TB infection (LTBI) and active TB disease [8].

Among direct diagnostic methods, microscopy—after acid-fast staining of clinical specimens—is a cornerstone for the identification of mycobacteria and TB due to its speed, cost-effectiveness, and accessibility. However, it has several limitations, particularly its lower sensitivity in certain patient populations, its inability to distinguish between MTBC and NTM, and its inability to provide data on drug resistance [22]. Hence, more advanced techniques, such as Nucleic Acid Amplification Tests (NAATs), which offer higher sensitivity and specificity, are often required to confirm the diagnosis—especially in TB smear-negative cases or in suspected drug-resistant strains—since they provide additional details about the infection and certain resistance-associated mutations. Usually, specific NAATs for NTM detection are not included in mycobacterial diagnostic workflows: NTM diagnosis is suspected in smear-positive NAAT-negative MTBC samples and confirmed by culture [23,24,25]. The culture of sputum or other clinical samples remains the gold standard for mycobacterial identification because mycobacteria grow on specialized media. According to WHO guidelines, it is important to use two media types: one liquid and one solid (e.g., BACTEC^TM^ MGIT^TM^ and Lowenstein–Jensen). The combined use of both media increases sensitivity, optimizing the recovery of different species of mycobacteria and achieving rates of 90–95% or higher in many cases [26,27]. The detection of *mycobacteria* in culture is crucial for correctly identifying the species and determining the susceptibility pattern; however, it could take up to 6–8 weeks for MTBC or slowly growing mycobacteria (SGM) to obtain a confirmatory result, which delays the beginning of the treatment, making it the primary limitation of this method [24]. A significant advancement in TB diagnosis is the application of molecular techniques, primarily polymerase chain reaction (PCR), directly on clinical specimens [22,28,29]. PCR, being highly specific and sensitive, enables the rapid detection of Mycobacterium tuberculosis DNA and, at the same time, allows the identification of drug-resistant strains, including multidrug-resistant and extensively drug-resistant TB [5,12].

In the past few years, the TB diagnostic workflow has been further transformed by the commercialization of a rapid molecular test, precisely Xpert^®^ MTB/RIF Ultra (GeneXpert, Cepheid, Sunnyvale, CA, USA). This extremely sensitive platform can detect MTBC DNAs in respiratory samples while assessing rifampicin resistance in a turnaround time of less than 2 h. This assay enables the rapid identification of TB-infected patients, which helps limit its transmission and permit appropriate antimicrobial treatment based on rifampicin resistance information [28,29].

Given the critical need for rapid and accurate diagnosis of TB and NTM to ensure effective disease management and reduce the global burden, this study aims to evaluate the diagnostic performance of the STANDARD™ M10 MTB/NTM assay (SD Biosensor, Inc., Gyeonggi, Republic of Korea). Building on previous studies evaluating molecular approaches for MTBC and NTM detection [30,31], we also propose potential fields of application within a hypothetical mycobacterial diagnostic workflow. This novel molecular assay is designed for the rapid detection and differentiation of MTBC and NTM, with the goal of enhancing etiological agent identification, enabling prompt initiation of appropriate antimicrobial therapy, reducing transmission risk, and preventing the development of drug-resistant strains.

## 2. Materials and Methods

### 2.1. Collection and Decontamination of Different Human Specimens

Between January 2023 and December 2024, 155 specimens were collected from patients suspected of having MTB/NTM infections. Samples were obtained from two microbiology and virology laboratories in north-western Italy: 120 (77.4%) from the University Hospital Città della Salute e della Scienza di Torino (Turin, Italy) and 35 (22.6%) from the Maggiore della Carità Hospital (Novara, Italy). The biological specimens were categorized as follows: sputum, bronchoalveolar lavage, exudate/pus, bronchial aspirate, biopsy, urine samples, gastric aspirate, cerebrospinal fluid, and pleural fluid. Notably, most samples were obtained from the respiratory tract. Each biological specimen underwent a range of microbiological analyses, including microscopy, culture, and molecular biology techniques.

**Figure 1:**
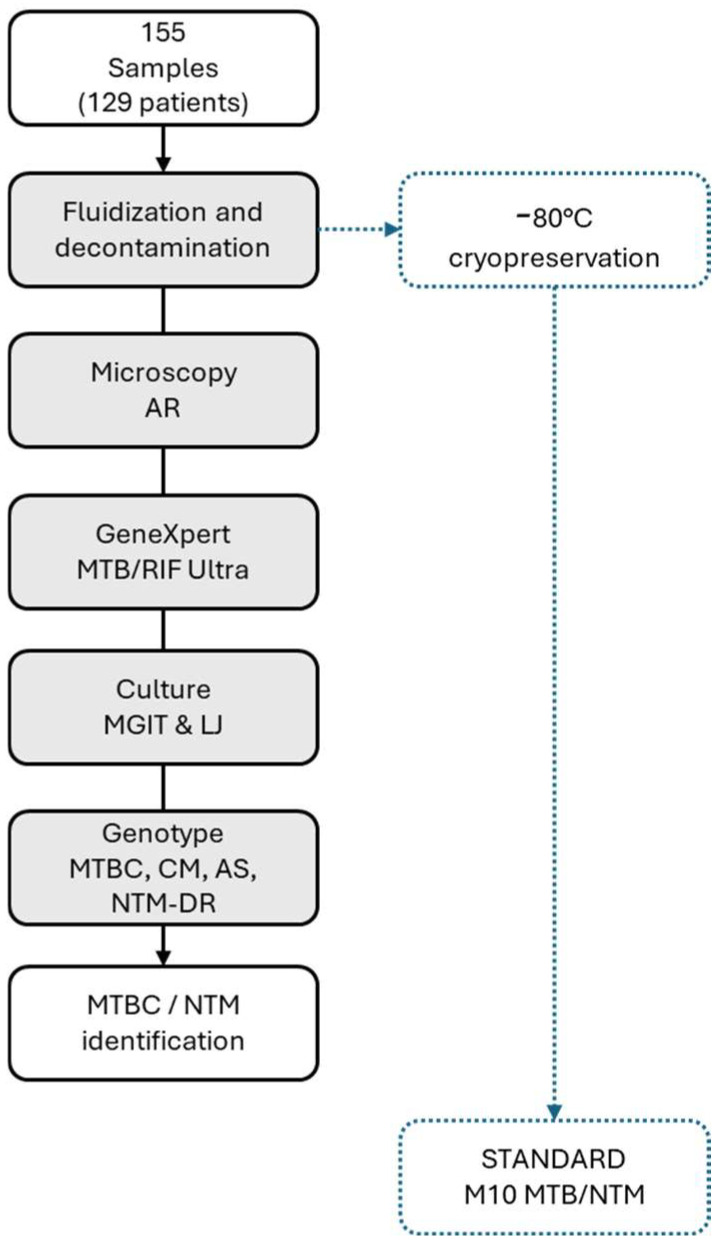
Study workflow: the standard of care is represented by gray rectangles and black arrows; the experimental arm of the study is represented by blue dotted-line rectangles and arrows.

Initially, the samples underwent chemical fluidization and decontamination with N-acetyl-L-cysteine (NALC) and sodium hydroxide (NaOH) according to the manufacturer’s instructions using MycoTB (Copan, Brescia, Italy). Briefly, 2.5 mL of each sample was combined with 2.5 mL of NALC/NaOH solution (containing 4% NaOH, 2.9% sodium citrate, and 0.5% NALC, resulting in a final NaOH concentration of 1%). After vortexing, the mixture was incubated at room temperature for 3 to 10 min, depending on the sample type and its contamination probability (e.g., 3 min for pleural fluid, 10 min for exudate/pus). Forty-five mL of phosphate-buffered saline (PBS, Copan) was added, and the suspension was concentrated by centrifugation at 3000 *g* for 10 min. Subsequently, the supernatant was discarded, and the pellet was resuspended in 2 mL of PBS. An aliquot (500 µL) of each decontaminated sample was retained and stored at −80 °C for prolonged preservation; the remaining aliquot was used for standard-of-care methods (SoC): microscopy, culture, and molecular assays [26,27]. A schematic representation of the study workflow is depicted in Figure 1.

### 2.2. Mycobacteria Staining and Culture

As regards microscopy, a drop of the decontaminated suspension was used to prepare a slide for automated auramine-rhodamine (AR) staining by BASO AS-316T (Va.Ni.Ca srl, Genova, Italy), which was examined under fluorescence microscopy at 200× magnification. The interpretative criteria—following CDC [32] and national recommendations (https://amcli.it/wp-content/uploads/2024/04/2023-18_TUBERCOLOSI-E-ALGORITMO-1.pdf, accessed on 31 January 2026)—are summarized in Table 1.

Mycobacterial culture was performed on both liquid and solid media according to international guidelines (https://www.ecdc.europa.eu/en/publications-data/handbook-tuberculosis-laboratory-diagnostic-methods-european-union-updated-2023, accessed on 30 May 2025).

In particular, 500 µL of the decontaminated suspension from the various biological specimens were inoculated into the liquid medium using the Mycobacteria Growth Indicator Tube (MGIT) 960 system (Becton, Dickinson and Company, Franklin Lakes, NJ, USA), and 250 µL were spread onto solid BBL Lowenstein–Jensen (LJ) medium (Becton, Dickinson and Company). Both media were incubated aerobically at 37 °C for up to 8 weeks.

Positive cultures (positivity signals by MGIT960 or visible colonies on LJ) were further confirmed by microscopic observation with the automated Ziehl–Neelsen (ZN) staining by AS-316GT (Va.Ni.Ca srl) instrument. Mycobacterial identification was performed using the Hain Lifescience GmbH (Nehren, Germany) Line Probe Assay (LPA) commercial system with GenoLyse v.1.0 and GenoType kits, according to manufacturer instructions. For MTBC, Genotype MTBC, and NTM, Genotype Mycobacterium CM v.2.0, GenoType Mycobacterium AS v.1.0, and GenoType NTM-DR v.2.0 were used, respectively.

### 2.3. GeneXpert MTB/RIF Ultra

GeneXpert MTB/RIF Ultra was performed on decontaminated respiratory specimens using a modified protocol, as the assay was applied to samples that had already been processed according to routine mycobacterial diagnostic procedures. As a molecular SoC method, CE-IVD GeneXpert MTB/RIF Ultra (ULTRA) (Cepheid, Sunnyvale, CA, USA) was used according to the manufacturer’s instructions. Briefly, 0.5 mL of each decontaminated respiratory specimen was mixed with 2.5 mL of Sample Reagent (Cepheid), vortexed, and incubated at room temperature for 15 min. Subsequently, 2 mL of the processed mixture were loaded into the ULTRA cartridge using the disposable transfer pipette provided with the kit. GeneXpert MTB/RIF Ultra is a fully automated system that performs nucleic acid extraction and real-time PCR for the detection of *M. tuberculosis* DNA and rifampicin resistance, providing results in approximately 80 min. Results were interpreted according to the manufacturer’s instructions using a semi-quantitative scale: negative, trace, very low, low, medium, and high [28].

### 2.4. STANDARD™ M10 Assay

For CE-IVD STANDARD™ M10 MTB/NTM (SD Biosensor) evaluation, 0.5 mL aliquots of decontaminated specimens were stored at −80 °C and subsequently thawed for testing. The aliquots were preserved for a maximum of four months before analysis, and no additional decontamination step was performed after thawing. The assay was performed according to the manufacturer’s instructions, except that frozen, previously decontaminated specimens were used. Briefly, 0.5 mL of the thawed aliquot was added to 1 mL of Pretreatment Solution (SD Biosensor), vortexed, and incubated at room temperature for 15 min. Subsequently, 1.4 mL of the processed mixture was transferred into the M10 cartridge using the STANDARD™ Disposable dropper provided in the kit. STANDARD™ M10 MTB/NTM is a fully automated real-time PCR assay for the detection and differentiation of MTBC and NTM, with results available in approximately 72 min. Although the kit is validated for sputum and sputum sediment, additional specimen types were tested to further investigate its performance and potential application on different biological matrices.

### 2.5. Statistical Analysis

Data were analyzed using GraphPad QuickCalcs (Dotmatics, Boston, MA, USA). The diagnostic performance of the different microbiological methods was assessed by calculating overall agreement, Cohen’s kappa coefficient (κ), sensitivity, and specificity. Cohen’s kappa coefficient (κ) was interpreted as follows: <0.20, poor; 0.21–0.40, fair; 0.41–0.60, moderate; 0.61–0.80, substantial; >0.80, almost perfect agreement. The culture method was used as the reference method.

## 3. Results

A total of 155 specimens were obtained from 129 patients with suspected MTBC/NTM infections, as summarized in Table 2. As expected, most samples originated from the respiratory tract (78.1%), including 58 sputum samples (47.9%), 47 bronchoalveolar lavage (BAL) samples (38.8%), 12 gastric aspirates (9.9%), and 4 bronchial aspirates (3.3%). The remaining 21.9% were collected from other body sites.

**Figure 2:**
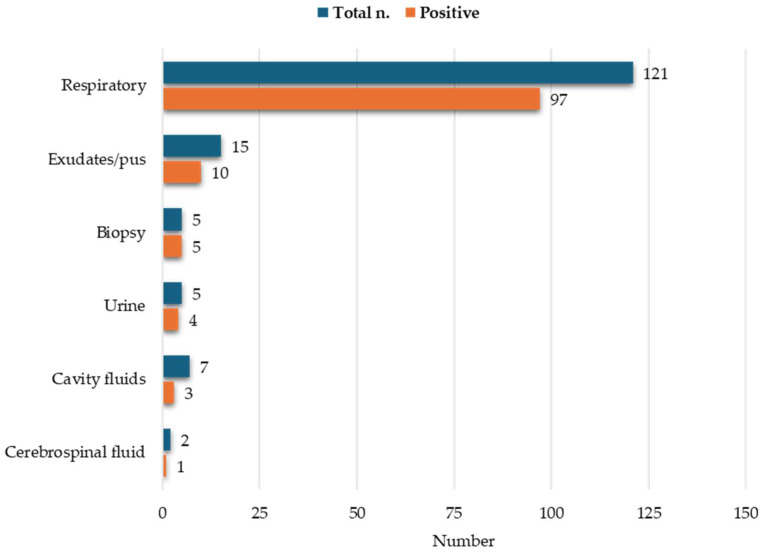
Number of collected specimens by type reported as positive (orange) and total (blue).

Culture positivity, the gold standard for mycobacterial detection, defined as bacterial growth in at least one liquid medium and in the solid medium, evaluated for MTBC and/or NTM, was obtained in 120 specimens, while 35 remained negative. The majority of positive samples were detected by both liquid and solid culture (94/120; 78.3%) and originated from the respiratory tract (97/120; 80.8%), with a balance between sputum (49/97; 50.5%) and samples obtained with invasive methods (Figure 2).

Table 3 reports the identification of all the mycobacterial strains—as species or group—obtained by Line Probe Assays (LPA). Briefly, MTBC was detected in 67 out of 124 culture-positive specimens (54%), predominantly *Mycobacterium tuberculosis*, which accounted for 64 out of 67 MTBC isolates (95.5%), while NTM were identified in the remaining 57 specimens (46%). We registered no failures in identifications made using LPA. Among NTM, most isolates (48/57, 84.2%) belonged to slow-growing mycobacteria (SGM), primarily *M. avium* and *M. intracellulare*, followed by rapid-growing mycobacteria (9/57, 15.8%) (RGM), mainly *M. abscessus* subsp. *abscessus*. Notably, two cases of coinfection were identified at the patient level: one involving *Mycobacterium simiae* and *Mycobacterium tuberculosis*, and one involving *Mycobacterium avium* and *Mycobacterium intracellulare*. In the latter case, the coinfection was detected in three different specimens from the same patient. Regarding genotypic antimicrobial susceptibility profiles, NTM-DR identified the *erm(41)* T28C mutation in two *Mycobacterium abscessus* subsp. *abscessus* isolates, whereas no mutations in the *rpoB* region were detected by Xpert^®^ MTB/RIF Ultra (Cepheid, Berkeley, CA, USA). Among NTM-positive specimens, most isolates were recovered from the respiratory tract (45/57, 78.9%), specifically 21 from sputum, 21 from BAL, and 2 from bronchial aspirate. The remaining 12 NTM isolates originated from exudate/pus (n = 6), urine (n = 4), and tissue samples (n = 2).

**Figure 3:**
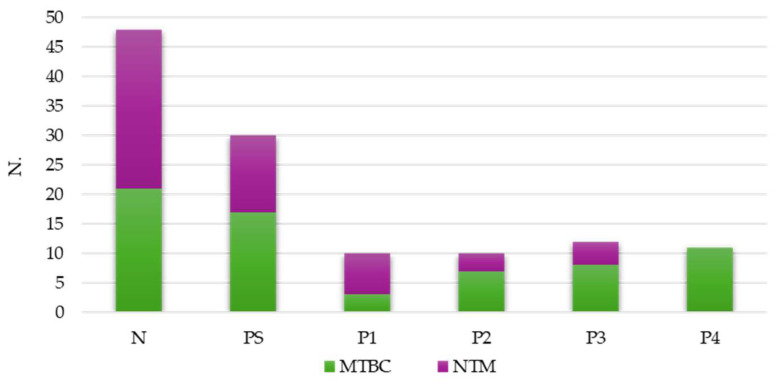
Microscopy results: number of negative and positive specimens reported as total, MTBC or NTM by fluorescence interpretative criteria (N: negative; PS: positive, scanty; P1: positive +; P2: positive ++; P3: positive +++; P4: positive ++++).

A preliminary sample examination by fluorescence microscopy was used and interpreted with semi-quantitative criteria. A total of 73 (47.1%) out of 155 specimens tested positive: the majority of these positive results were classified as PS (41.1%), comparable frequencies were observed for categories P1 and P2 (13.7%) and at the P4 level (15.1%), only MTBC was detected (Figure 3).

When fluorescence microscopy and culture were compared, a fair agreement (67.7%) was observed (Cohen’s kappa 0.362; C.I. 95% 0.241 to 0.482), with microscopy showing an overall sensitivity and specificity at 60.8% and 91.4%, respectively. Additionally, the comparison, grouped by MTB or NTM positivity, revealed different results as shown in Table 4. Notably, moderate agreement (76.5%) was observed only for MTB (Cohen’s kappa of 0.535 for MTBC vs. 0.370 for NTM), but the difference was statistically significant. In contrast, the association between culture and the molecular assays—specifically GeneXpert MTB/RIF Ultra and STANDARD™ M10 MTB/NTM—showed different levels of agreement and sensitivity, even though 100% specificity was consistently observed. Considering GeneXpert MTB/RIF Ultra (only for MTBC), the agreement with culture was 98.1%, with high levels of specificity and sensitivity (96%; in detail, 100% when microscopy+/culture+ and 83.3% when microscopy-/culture+). Furthermore, when STANDARD™ M10 MTB/NTM was compared with culture specifically for NTM, the Cohen’s kappa coefficient was 0.566, indicating moderate agreement, with reduced sensitivity (50%). Since most of the specimens were from the respiratory tract, a further agreement analysis was performed comparing the results obtained with Xpert^®^ MTB/RIF Ultra and STANDARD™ M10 MTB/NTM. Considering the Xpert^®^ MTB/RIF Ultra, the results (calculated only for the MTBC) indicated high agreement (98.1%; C.I. 95% 93.0 to 99.0) with Cohen’s kappa coefficients of 0.962 (C.I. 95% 0.911 to 1.000), and a sensitivity of 96.0% (C.I. 95% 85.8 to 99.7). On the other hand, STANDARD™ M10 MTB/NTM showed high specificity (100%) but lower values of agreement (77.7%; C.I. 95% 69.4 to 84.2) with a Cohen’s kappa coefficient of 0.507 (C.I. 95% 0.364 to 0.650) and sensitivity 72.2% (C.I. 95% 62.5 to 80.2).

As regards coinfections and STANDARD™ M10 MTB/NTM results, of the three positive samples for *M. avium* and *M. intracellulare*, only one was identified as NTM; the others were negative. Considering the single sample positive for MTBC and NTM, STANDARD™ M10 MTB/NTM missed the MTBC-positive result, identifying only NTM.

**Figure 4:**
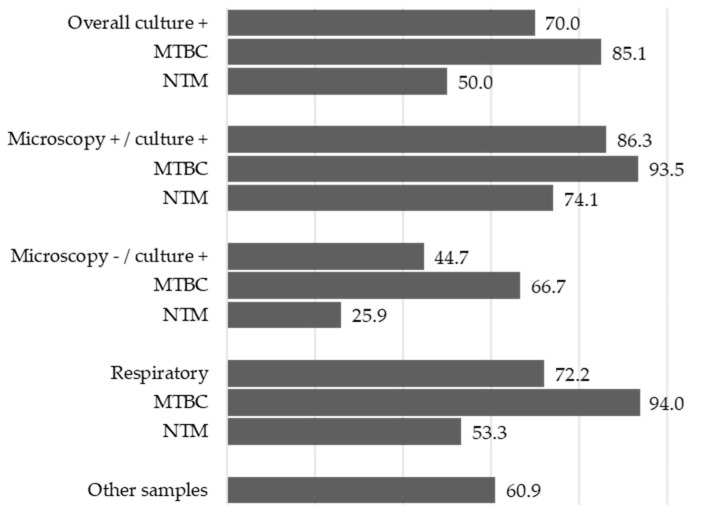
Graphical representation of the percentage of STANDARD™ M10 MTB/NTM sensitivity stratified for microscopy and culture results, mycobacteria group, and sample type.

Figure 4 focuses on the sensitivity of the STANDARD™ M10 MTB/NTM, which is stratified according to different conditions. The sensitivity value was higher for MTBC than for NTM in all conditions, with better performance observed in respiratory samples. Finally, when microscopy was negative, and culture was positive, sensitivity decreased to 44.7%, particularly to 66.7% for MTBC and 25.9% for NTM.

## 4. Discussion

Despite recent advances in the diagnostic routine for mycobacterial isolation and identification, a clinically relevant gap remains in the accurate differentiation between the MTBC and NTM [16,33]. The latter includes numerous environmental species and groups of mycobacteria that are increasingly associated with human infections, particularly in specific patient populations such as immunocompromised individuals, patients with cystic fibrosis, those with structural lung diseases, transplant recipients, and the elderly [15,18,34,35,36]. In these patients, mycobacterial infection can lead to a poor prognosis, resulting in high rates of morbidity and mortality, with severe respiratory infections accounting for the vast majority of cases [34,37,38,39].

The standard protocol follows a systematic workflow that begins with microscopic analysis using acid-fast staining or fluorescence. This is followed by culture on liquid and/or solid media. Finally, when the laboratory is appropriately equipped, molecular assays are performed not only to determine whether a specimen is positive or negative, but also to identify the specific mycobacterial species or group. As both MTBC and NTM can be causative agents of infection, accurate differentiation between them is essential for appropriate clinical management [8,16,22,40].

Against the background of the increasing burden of NTM infections and the persistent challenges in MTBC/NTM differentiation, this study aims to contribute by evaluating the performance of a rapid molecular assay specifically designed for early etiological discrimination in routine clinical specimens [17]. In this multicenter study, the diagnostic performances of conventional and innovative techniques for MTBC and NTM detection in various biological specimens were evaluated in two large microbiology and virology laboratories of north-western Italy: the University Hospital Città della Salute e della Scienza di Torino (Turin) and the Maggiore della Carità Hospital (Novara). Between these two centers, according to the national recommendations, the pre-analytical and analytical phases were comparable as stated in the Materials and Methods section. The STANDARD™ M10 MTB/NTM performance assessment was the main goal of our study in comparison with our standard of care and, in particular, with culture used as reference.

Although culture is regarded as the gold standard method, it is time-consuming and susceptible to failure to identify NTM, primarily due to specific culture conditions, such as culture media, incubation time, and temperature. On liquid media, for example, mycobacteria detection is usually more rapid, but some NTM are unable to grow. On the other hand, solid media are typically examined every 7 days, which can delay detection of positivity. Moreover, the prolonged incubation period carries a significant risk of contamination, despite the use of standard decontamination protocols [23]. While microscopy can provide an early indication of positivity in various specimens, it has notable limitations, including a higher risk of false negatives, especially for acid-fast staining, and the inability to distinguish between MTBC and NTM. In this study, the culture positivity rates for MTBC and NTM were 54% and 46%, respectively. Even though respiratory specimens were the most frequently collected and tested positive for both types of mycobacteria, 21.9% of non-respiratory samples were also processed to evaluate performance across different biological matrices. Culture detected 120 positive specimens, whereas fluorescence microscopy identified a lower number of positives—specifically, 76 samples. Fluorescent microscopy showed an overall sensitivity and specificity of 60.8% and 91.4%, respectively. Notably, a moderate agreement between culture and microscopy was observed only for MTB (Cohen’s kappa 0.535 culture for MTBC vs. Cohen’s kappa 0.370 culture for NTM).

The next step was to assess the performance of two different molecular approaches for mycobacterial detection, using culture as the reference method. Notably, GeneXpert MTB/RIF Ultra can provide rapid detection of MTBC, with results in 2 h, along with rifamycin resistance profiling. In contrast, the STANDARD™ M10 MTB/NTM allows clear differentiation between MTBC and NTM, but does not provide information on NTM species discrimination or MTBC antimicrobial resistance. When the two methods were compared for MTBC identification, using culture as the reference method, a strong agreement for both was observed (Cohen’s kappa coefficients were 0.962 for GeneXpert MTB/RIF Ultra and 0.866 for STANDARD™ M10 MTB/NTM), with >85% and 100% of sensitivity and specificity, respectively. These outcomes confirmed a strong performance of the molecular approaches. GeneXpert MTB/RIF Ultra also provided a quantitative classification of MTBC-positive results. The outcomes showed that most specimens were categorized as either PL (~38%) or PH (~31%), while the remaining were distributed among PT, PVL, and PM. For STANDARD™ M10 MTB/NTM, concordance with culture for NTM was analyzed, demonstrating moderate agreement, with 50% sensitivity and 100% specificity. Notably, the diagnostic sensitivity for NTM decreased markedly in microscopy-negative/culture-positive specimens, to 25.9%, highlighting an important limitation of the assay in paucibacillary samples.

Other authors evaluated the STANDARD™ M10 MTB/NTM assay on liquid cultures positive for MTBC or NTM, reporting full concordance for MTBC-positive samples (50/50), slightly lower agreement for NTM-positive cultures (47/50), and 100% specificity [30]. Additionally, Luukinen et al. (2024) compared culture, GeneXpert Ultra, and M10 MTB/NTM across various specimen types. Overall, the results of the present study are in line with previous reports evaluating the STANDARD™ M10 MTB/NTM assay in low-TB-incidence settings, confirming its high specificity for MTBC detection and lower sensitivity for NTM, particularly in paucibacillary specimens [30,31]. For MTBC detection, GeneXpert Ultra showed a sensitivity of 88.9% and a specificity of 97.4%, while STANDARD™ M10 MTB/NTM demonstrated a sensitivity of 84.4% and a specificity of 98.3%. In contrast, as also observed in our study, a lower sensitivity was reported for NTM detection, with a value of 65.7% [31]. Notably, despite some similarities between our study and previous research, there is currently no literature evidence of a comparative assessment that examines the entire workflow—from microscopy to molecular methods—through an integrated, comparative analysis.

Analyzing STANDARD™ M10 MTB/NTM results and coinfections, the cases of undetected MTBC, in the MTBC/NTM positive sample, and of NTM, in the two *M. avium* and *M. intracellulare* positive samples, occurred in microscopic negative and scanty positive specimens, respectively. These results could be justified by the number of bacilli/mL, which is near the limit of detection. Moreover, since fluorochrome staining, used in our study, is more sensitive and slightly less specific than carbol fuchsine staining, the high performance of the STANDARD™ M10 MTB/NTM could be mitigated in the microscopy+/culture+ group, particularly in samples with low mycobacterial load. In settings using carbol fuchsine staining, STANDARD™ M10 MTB/NTM performance could be more effective. In these cases, sensitivity in microscopy-/culture+ samples could be enhanced, allowing interception of more MTBC or NTM-positive samples in advance. On the other hand, considering not only sensitivity but also STANDARD™ M10 MTB/NTM specificity, in settings with a limited number of operators expert in the microscopic diagnosis of Mycobacteria, e.g., in spoke laboratories or emergency settings, its application could limit false-positive and false-negative results. Considering our results, the STANDARD™ M10 MTB/NTM assay could be used in specific settings, such as when microscopy is negative, and samples are collected from a sterile anatomic region. A positive result on these specimens is highly suggestive of NTM disease and can precede culture positivity. It should be emphasized that, unlike GeneXpert MTB/RIF Ultra, the STANDARD™ M10 MTB/NTM assay does not provide information on MTBC drug resistance. Therefore, in cases of MTBC detection, additional molecular testing remains necessary to guide appropriate antimicrobial therapy.

Moreover, the application of the STANDARD™ M10 MTB/NTM assay could be reserved for patients with a high clinical suspicion of NTM infection, especially immunocompromised patients.

This is the case, for example, in lung transplant recipients, due to the increased risk of developing a clinically relevant mycobacterial infection, which has been shown to impact outcomes in the context of immunosuppressive treatment [41,42]. In this population, early detection of possible NTM/MTBC colonization or infection, even in the absence of precise differentiation, may help guide the intensity of immunosuppressive therapy [43]. In particular, it may support withholding high-dose steroid treatment in cases of rejection where it is not strictly necessary (e.g., A1 acute cellular rejection), while awaiting definitive microbiological results for etiological confirmation and species identification [44]. Vice versa, decisions in favor of a more aggressive immunosuppressive regimen can be supported by an additional negative mycobacterial test.

Applying the assay in these selective cases, alongside other standard tests, could provide preliminary data many days before the culture results are available. However, this assay cannot identify NTM, which is fundamental for defining appropriate antibiotic therapy when and if necessary. To overcome this limit, rapid molecular assays are under development to enable NTM identification, particularly for respiratory samples. In addition, next-generation sequencing methods are increasingly being adopted in microbiology laboratories and enable both mycobacterial species identification and the provision of useful information on antimicrobial susceptibility profiles. However, their high costs and longer turnaround times currently limit their practical advantages for direct use on clinical samples.

Taking these considerations into account, a possible application of the STANDARD™ M10 MTB/NTM assay in the mycobacterial diagnostic workflow could be as a screening test for patients with a high suspicion of mycobacterial infection. However, for MTBC detection, it should be noted that STANDARD™ M10 MTB/NTM has shown lower sensitivity than GeneXpert MTB/RIF Ultra, especially in microscopy-negative samples. Moreover, if MTBC is positive, an additional molecular test should be performed to evaluate rifampicin resistance.

This study has some limitations. The sample size was relatively limited, and most specimens were respiratory samples, which may affect the generalizability of the results to other clinical settings. In addition, the STANDARD™ M10 MTB/NTM assay was not designed to provide species-level identification of NTM or information on MTBC drug resistance. However, the multicenter design of the study, the inclusion of a heterogeneous set of clinical specimens, and the comparison with standard diagnostic methods in routine clinical practice represent key strengths of this work.

## 5. Conclusions

Our study reiterates the central role of culture, which remains the gold standard for mycobacterial diagnosis, although MTB/NTM molecular tools have significantly reduced the turnaround time for laboratory results. In recent years, molecular tests have revolutionized the diagnosis of mycobacterial infections; however, they must be used in conjunction with other techniques, as there is always a trade-off between the advantages and limitations of each assay. Based on our results, to optimize the use of the STANDARD™ M10 MTB/NTM assay, we propose its application to selected samples suggestive of MTBC/NTM disease, or to patients with a high likelihood of NTM infection, particularly in clinical settings where a rapid etiological orientation may support therapeutic decisions or the modulation of immunosuppressive therapy.

## Figures and Tables

**Table 1:** Interpretative criteria of auramine-rhodamine staining for acid-fast bacteria (AFB).

Number of AFB/Fields	Interpretation	Abbreviation
0	Negative	N
1–2/30 fields	Positive (scanty)	PS
1–9/10 fields	Positive (+)	P1
1–9/field	Positive (++)	P2
10–90/field	Positive (+++)	P3
>90/field	Positive (++++)	P4

**Abbreviations:** P1, P2, P3, and P4 correspond to 1+, 2+, 3+, and 4+ positivity grades, respectively, indicating increasing AFB density as determined by fluorescence microscopy.

**Table 2:** Details of the different sample types, presented as numbers and percentages.

Sample Type	N.	%
Total samples	155	-
Respiratory	121	78.1
Exudates/pus	15	9.7
Cavity fluids	7	4.5
Biopsies	5	3.2
Urine	5	3.2
Cerebrospinal fluids	2	1.3

**Table 3:** Species/group of the mycobacteria recovered from the different specimens.

Species/Group	N.	%
MTBC	67	54.0
M. tuberculosis *	64	51.6
*M. bovis BCG*	3	2.4
NTM	57	46.0
SGM	48	38.7
*M. avium ^#^*	17	13.7
*M. intracellulare ^#^*	17	13.7
*M. chimaera*	5	4.0
*M. gordonae*	3	2.4
*M. xenopi*	2	1.6
M. simiae *	2	1.6
*M. marinum*	1	0.8
*M. parascrofulaceum*	1	0.8
RGM	9	7.3
*M. abscessus* subsp. *abscessus*	5	4.0
*M. chelonae*	2	1.6
*M. fortuitum group*	1	0.8
*M. mucogenicum*	1	0.8

* *M. simiae* and *M. tuberculosis* coinfection (n = 1) ^#^ *M. avium* and *M. intracellulare coinfection* (n = 3). Abbreviations: *Mycobacterium tuberculosis* complex, MTBC; non-tuberculous mycobacteria, NTM; rapid-growing mycobacteria, RGM; slow-growing mycobacteria, SGM.

**Table 4:** Overall comparison between fluorescence microscopy. Xpert^®^ MTB/RIF Ultra and STANDARD™ M10 MTB/NTM with culture reference method (**A**) and stratified for MTBC (**B**), NTM (**C**), and respiratory samples (**D**).

**(A) Overall**					
**Test**	**N.**	**Agreement**	**Cohen’s Kappa**	**Sensitivity**	**Specificity**
Fluorescence microscopy	155	67.7 (60.0–74.6)	0.362 (0.241–0.482)	60.8 (51.9–69.1)	91.4 (76.9–97.8)
STANDARD™ M10 MTB/NTM	155	76.8 (69.5–82.8)	0.513 (0.391–0.635)	70.0 (61.3–77.5)	100 (8.2–100)
**(B) MTBC**					
**Test**	**N.**	**Agreement**	**Cohen’s Kappa**	**Sensitivity**	**Specificity**
Fluorescence microscopy	155	76.5 (67.3–83.7)	0.535 (0.383–0.688)	68.7 (56.8–78.5)	91.4 (76.9–97.8)
Xpert^®^ MTB/RIF Ultra	107	98.1 (93.0–99.9)	0.962 (0.911–1.000)	96.0 (85.8–99.7) *	100 (92.5–100)
STANDARD™ M10 MTB/NTM	155	93.6 (88.4–96.6)	0.866 (0.787–0.946)	85.1 (74.5–91.9)	100 (95.0–100)
**(C) NTM**					
**Test**	**N.**	**Agreement**	**Cohen’s Kappa**	**Sensitivity**	**Specificity**
Fluorescence microscopy	155	66.3 (56.0–75.3)	0.370 (0.210–0.530)	50.0 (37.1–62.9)	91.4 (76.9–97.8)
STANDARD™ M10 MTB/NTM	155	82.6 (75.8–87.8)	0.566 (0.432–0.700)	50.0 (37.1–62.9)	100 (95.6–100)
**(D) Respiratory Samples**					
**Test**	**N.**	**Agreement**	**Cohen’s Kappa**	**Sensitivity**	**Specificity**
Fluorescence microscopy (AR)	121	69.4 (59.0–75.5)	0.353 (0.208–0.498)	65.0 (55.0–73.7)	87.5 (68.2–96.5)
Xpert^®^ MTB/RIF Ultra	107	98.1 ** (93.0–99.9)	0.962 ** (0.911–1.000)	96.0 ** (85.8–99.7)	100 ** (92.5–100)
STANDARD™ M10 MTB/NTM	121	77.7 (69.4–84.2)	0.507 (0.364–0.650)	72.2 (62.5–80.2)	100 (83.7–100)

* in case of microscopy+/culture+, sensitivity: 100%; in case of microscopy-/culture+, sensitivity: 83.3%. ** MTBC only.

## Data Availability

The original contributions presented in this study are included in the article. Further inquiries can be directed to the corresponding author.
